# Occlusion of the Superior Mesenteric Artery in Rats Reversed by Collateral Pathways Activation: Gastric Pentadecapeptide BPC 157 Therapy Counteracts Multiple Organ Dysfunction Syndrome; Intracranial, Portal, and Caval Hypertension; and Aortal Hypotension

**DOI:** 10.3390/biomedicines9060609

**Published:** 2021-05-26

**Authors:** Mario Knezevic, Slaven Gojkovic, Ivan Krezic, Helena Zizek, Dominik Malekinusic, Borna Vrdoljak, Hrvoje Vranes, Tamara Knezevic, Ivan Barisic, Katarina Horvat Pavlov, Domagoj Drmic, Miro Staroveski, Antonija Djuzel, Zoran Rajkovic, Toni Kolak, Ivica Kocman, Eva Lovric, Marija Milavic, Suncana Sikiric, Ante Tvrdeic, Leonardo Patrlj, Sanja Strbe, Antonio Kokot, Alenka Boban Blagaic, Anita Skrtic, Sven Seiwerth, Predrag Sikiric

**Affiliations:** 1 Department of Pharmacology, School of Medicine, University of Zagreb, 10000 Zagreb, Croatia; mariknezevic@gmail.com (M.K.); slaven.gojkovic.007@gmail.com (S.G.); ivankrezic94@gmail.com (I.K.); zizekhelena@gmail.com (H.Z.); dominikmalekinusic@gmail.com (D.M.); borna.vrdoljak@gmail.com (B.V.); hrvoje.vranes@gmail.com (H.V.); 0101tamara@gmail.com (T.K.); inbarisic@gmail.com (I.B.); iddrmic@mef.hr (D.D.); miro.staroveski@gmail.com (M.S.); antonija.djuzel@gmail.com (A.D.); tkolak@kbd.hr (T.K.); kocman.ivica@gmail.com (I.K.); ante.tvrdeic@mef.hr (A.T.); patrljleo@gmail.com (L.P.); strbes@gmail.com (S.S.); abblagaic@mef.hr (A.B.B.); 2 Department of Pathology, School of Medicine, University of Zagreb, 10000 Zagreb, Croatia; katarina.horvat@gmail.com (K.H.P.); eva.lovric@kb-merkur.hr (E.L.); marija.milavic@mef.hr (M.M.); suncanasikiric@gmail.com (S.S.); skrtic.anita@gmail.com (A.S.); sven.seiwerth@mef.hr (S.S.); 3 Department of Surgery, Faculty of Dental Medicine and Health, University of Osijek, 31000 Osijek, Croatia; zrajkovi@net.hr; 4 Department of Anatomy and Neuroscience, School of Medicine, Josip Juraj Strossmayer University of Osijek, 31000 Osijek, Croatia; antonio.kokot@mefos.hr

**Keywords:** BPC 157, superior mesenteric artery occlusion, vascular recruitment, rats

## Abstract

Gastric pentadecapeptide BPC 157 therapy counteracts multiple organ dysfunction syndrome in rats, which have permanent occlusion of the superior mesenteric artery close to the abdominal aorta. Previously, when confronted with major vessel occlusion, its effect would rapidly activate collateral vessel pathways and resolve major venous occlusion syndromes (Pringle maneuver ischemia, reperfusion, Budd–Chiari syndrome) in rats. This would overwhelm superior mesenteric artery permanent occlusion, and result in local, peripheral, and central disturbances. **Methods:** Assessments, for 30 min (gross recording, angiography, ECG, pressure, microscopy, biochemistry, and oxidative stress), included the portal hypertension, caval hypertension, and aortal hypotension, and centrally, the superior sagittal sinus hypertension; systemic arterial and venous thrombosis; ECG disturbances; MDA-tissue increase; and multiple organ lesions and disturbances, including the heart, lung, liver, kidney, and gastrointestinal tract, in particular, as well as brain (cortex (cerebral, cerebellar), hypothalamus/thalamus, hippocampus). BPC 157 therapy (/kg, abdominal bath) (10 µg, 10 ng) was given for a 1-min ligation time. **Results:** BPC 157 rapidly recruits collateral vessels (inferior anterior pancreaticoduodenal artery and inferior mesenteric artery) that circumvent occlusion and ascertains blood flow distant from the occlusion in the superior mesenteric artery. Portal and caval hypertension, aortal hypotension, and, centrally, superior sagittal sinus hypertension were attenuated or eliminated, and ECG disturbances markedly mitigated. BPC 157 therapy almost annihilated venous and arterial thrombosis. Multiple organ lesions and disturbances (i.e., heart, lung, liver, and gastrointestinal tract, in particular, as well as brain) were largely attenuated. **Conclusions**: Rats with superior mesenteric artery occlusion may additionally undergo BPC 157 therapy as full counteraction of vascular occlusion-induced multiple organ dysfunction syndrome.

## 1. Introduction

We have focused on the superior mesenteric artery occlusion and the stable gastric pentadecapeptide BPC 157. With the recovery of the rats, which have permanent occlusion of the superior mesenteric artery, we have attempted to introduce the stable pentadecapeptide BPC 157 therapy [1,2,3,4,5,6,7,8,9,10,11,12,13,14,15,16,17]. Confronted with major vessel occlusion [18,19,20,21,22,23,24,25], its effect rapidly activated collateral vessel pathways and re-established blood flow [18,19,20,21,22,23,24,25]. We hypothesized that this effect would overwhelm superior mesenteric artery permanent occlusion, and consequent local, peripheral, and central disturbances.

BPC 157 is known to have a very safe profile when used in ulcerative colitis. In a multiple sclerosis trial, a lethal dose (LD1) was not achieved [1,2,3,4,5,6,7,8,9,10,11,12,13,14,15,16,17]. As a cytoprotective agent [1,2,3,4,5,6,7,8,9,10,11,12,13,14,15,16,17], and a likely mediator of Robert’s cytoprotection [26], BPC 157 shows its effectiveness in the whole gastrointestinal tract, and counteracts various lesion models, exhibiting a particular effect on blood vessels [1,2,3,4,5,6,7,8,9,10,11,12,13,14,15,16,17].

Particularly noteworthy, we suggested that the essential point of recovery from occlusion of the superior mesenteric artery through activation of the collateral pathways to the bridge occlusion site [18,19,20,21,22,23,24,25] is manifestation of the activated Robert’s cytoprotection course [26]. This essential endothelium protection [27,28] was long ago implemented as the crucial class activity of cytoprotective agents [27,28], and BPC 157, in particular [29] (i.e., BPC 157 directly protected endothelium [2,29]). Consequently, BPC 157 prevents and reverses thrombosis formation after abdominal anastomosis [30], and major vessel occlusion [19,24,25]. Further, in general, BPC 157 alleviates peripheral vascular occlusion disturbances [18,19,20,21,22,23,24,25], and consequently various major venous occlusion syndromes (inferior caval vein syndrome, Pringle maneuver, ischemia, reperfusion, Budd–Chiari syndrome) [19,24,25]. BPC 157 therapy rapidly activates alternative bypassing pathways [18,19,20,21,22,23,24,25]. BPC 157′s endothelium point is strongly supported by its large interaction with the NO system (for a review, see [7]), and the most recent demonstration that BPC 157’s effect on vasomotor tone is carried out through BPC 157-specific activation of the Src-Caveolin-1-endothelial nitric oxide synthase (eNOS) pathway [31].

Thus, these findings [18,19,20,21,22,23,24,25] favor the recovering effect on the syndrome induced by permanent occlusion of a major artery (i.e., superior mesenteric artery), which has not been investigated yet. Likewise, after bilateral clamping of the common carotid arteries [20], given in reperfusion, BPC 157 counteracts stroke (i.e., both early and delayed neural hippocampal damage), achieving full functional recovery (Morris water maze test, inclined beam-walking test, lateral push test). Thus, recovery from peripheral vessel permanent occlusion-induced concomitant central disturbances, which have still not been investigated, may also be a likely effect of BPC 157 therapy.

Commonly, mesenteric vascular occlusion in the rat, as a model used frequently to study ischemic injury to the intestine (i.e., [32,33,34,35,36,37]), acknowledges intestinal ischemic injury as a life-threatening condition that requires immediate attention and treatment of any potential underlying etiology, and then, distant organ damage (multiple organ dysfunction) [38], which, however, remains less discussed [32,33,34,35,36,37]. However, each of these studies favors specific management of the superior mesenteric artery injuries, with success depending on the agent’s specific target. These targets in the supposed main underlying disturbance (i.e., immune/inflammatory cellular reactions) [32,33,34,35,36,37] may be quite distinctive, i.e., 5-HT1B and 5-HT1D receptors (sumatriptan) [32], PARα receptors (fenofibrate) [33], toll-like receptors (TLRs) (N-acetylserotonin (NAS) [34], T cells (cyclosporine and rapamycin) [35], neutrophils (i.e., quercin) [36], and melatonin [37]. Contrarily, the prime cause, the occlusion of the superior mesenteric artery itself, has remained largely ignored [32,33,34,35,36,37]. Additionally, in these studies, the success of the beneficial effect on gastrointestinal lesion recovery was not confirmed with recovery of the distant organ damage, peripherally and centrally, which were not investigated [32,33,34,35,36,37].

Thus, this novel general point of the rapid activation of collateral circulation to bypass and reestablish blood flow [18,19,20,21,22,23,24,25] may be a challenging resolving point in rats that have superior mesenteric artery occlusion, and may have, with therapy, a rapidly activated alternative specific bypassing pathway. Illustratively, we first reported the particular rapid activation of a bypassing loop from existing vessels (i.e., intestine arcade vessels network or left ovarian vein) [18,20]. This occurs in rats that have excluded part of the left colic artery and vein by two ligations, the ischemic/reperfusion colitis, or have infrarenal ligation of the inferior vena cava [18,20]. Furthermore, counteraction of the vascular occlusion disturbances was considered to be related to relieving a Virchow’s triad situation that may be commonly presented (i.e., duodenal venous congestion lesions [21], perforated cecum [22], bile duct ligation-induced liver cirrhosis and portal hypertension [23], Pringle maneuver, ischemia, reperfusion [25], and suprahepatic occlusion of the inferior caval vein (Budd–Chiari syndrome)) [24]. Consequently, while counteracting venous occlusion syndromes, BPC 157 counteracts severe venous hypertension (portal and caval) and aortal hypotension and ECG disturbances, progressing venous and arterial thrombosis, and multiple organ lesions in the lung, liver, kidney, and gastrointestinal tract [19,21,22,23,24,25]. Additionally, BPC 157 may be advantageous in precluding reperfusion-induced free radical injury [18,19,21,22,25,39,40,41].

Thus, in superior mesenteric artery occlusion in rats, the inferior anterior pancreaticoduodenal artery and inferior mesenteric artery may be bypassing vessels that are rapidly recruited. Consequently, we will demonstrate a similar rapid therapy effect in the emergency of occluded superior mesenteric artery-induced syndrome, comparable to the therapy effect of administration of the stable gastric pentadecapeptide BPC 157 in rat inferior caval vein syndrome [19], Pringle maneuver, ischemia, reperfusion [25], and Budd–Chiari syndrome [24]. Therefore, in a comparable life-threatening syndrome that occurs rapidly in rats with an occluded superior mesenteric artery, the therapy would also counteract this rapidly. This includes portal and inferior caval vein hypertension, abdominal aorta hypotension, and centrally, hypertension in the superior sagittal sinus (and thereby, rapid brain swelling), leading to severe venous hypertension and aortal hypotension, ECG disturbances, progressing venous and arterial thrombosis, and multiple organ lesions in the lung, liver, kidney, and gastrointestinal tract, in particular, as well as the brain. Finally, as a membrane stabilizer [16], BPC 157’s curative effect is achieved through its interaction with several molecular pathways [15,16,19,20,42,43,44,45,46,47,48].

## 2. Materials and Methods

### 2.1. Animals

This study was conducted with 12-week-old, 200-g body weight, male albino Wistar rats, randomly assigned at 6 rats/group/interval. Rats were bred in-house at the pharmacology animal facility, School of Medicine, Zagreb, Croatia. The animal facility was registered by the Directorate of Veterinary (Reg. No: HR-POK-007). Laboratory rats were acclimated for five days and randomly assigned to their respective treatment groups. Laboratory animals were housed in polycarbonate (PC) cages, 3 rats per cage, under conventional laboratory conditions at 20–24 °C, a relative humidity of 40–70%, and noise level of 60 dB. Each cage was identified with dates, the number of the study, group, dose, and the number and sex of each animal. Fluorescent lighting provided illumination for 12 h per day. A standard good laboratory practice (GLP) diet and fresh water was provided ad libitum. Animal care was in compliance with standard operating procedures (SOPs) of the pharmacology animal facility, and the European Convention for the Protection of Vertebrate Animals used for Experimental and other Scientific Purposes (ETS 123).

This study was approved by the local ethics committee (case number 380-59-10106-17-100/290). The ethical principles of the study complied with the European Directive 010/63/E, the Law on Amendments to the Animal Protection Act (Official Gazette 37/13), the Animal Protection Act (Official Gazette 135/06), the Ordinance on the protection of animals used for scientific purposes (Official Gazette 55/13), Federation of European Laboratory Animal Science Associations (FELASA) recommendations, and the recommendations of the Ethics Committee of the School of Medicine, University of Zagreb. The experiments were assessed by observers blinded as to the treatment.

### 2.2. Drugs

Medication was administered as described previously in vessel-occlusion studies [18,19,20,21,22,23,24,25], without the use of a carrier or peptidase inhibitor, for stable gastric pentadecapeptide BPC 157, a partial sequence of the human gastric juice protein BPC, which was freely soluble in water at pH 7.0 and in saline. BPC 157 (GEPPPGKPADDAGLV, molecular weight 1419; Diagen, Ljubljana Smartno, Slovenia) was prepared as a peptide with 99% high-performance liquid chromatography (HPLC) purity, with 1-des-Gly peptide being the main impurity. The dose and application regimens were as described previously [18,19,20,21,22,23,24,25].

### 2.3. Experimental Protocol

We performed ligation of the superior mesenteric artery at 2 mm from the outflow from the abdominal aorta in the anesthetized rats (intraperitoneally injected 40 mg/kg thiopental (Rotexmedica, Trittau, Germany) and 10 mg/kg diazepam (Apaurin; Krka, Slovenia)). Thereby, permanent occlusion by ligation of the superior mesenteric artery led to permanent alteration of blood flow, and a continuously progressing course.

All rats with an occluded superior mesenteric artery were sacrificed after 30 min of ligation, and medication was given after 1 min of ligation: 10 µg/kg BPC 157, 10 ng/kg BPC 157, or 5mL/kg saline. It was given intraperitoneally as 1 mL/rat abdominal bath.

For angiography, medication (10 µg/kg BPC 157, 10 ng/kg BPC 157 or 5 mL/kg saline) was applied intraperitoneally, as 1 mL/rat abdominal bath, after 15 min of ligation just before angiography.

**Figure 1:**
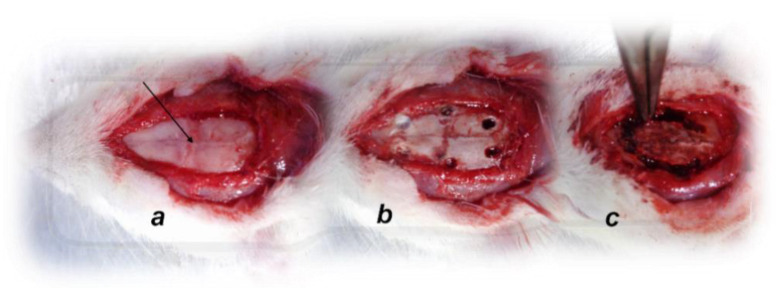
Animal preparation for the assessment procedure. (**a**). For superior sagittal sinus pressure recording, a single burr hole was placed in the rostral part of the sagittal suture, above the superior sagittal sinus (arrow); (**b**). Six burr holes drilled in three horizontal lines, with all of them medial to the superior temporal lines and temporalis muscle attachments; (**c**). Complete calvariectomy.

Recording of brain swelling was performed in rats at 15 min after the complete calvariectomy, and recording with a camera attached to a VMS-004 Discovery Deluxe USB microscope (Veho, Denver, CO, USA) was performed. Briefly, six burr holes were drilled in three horizontal lines, with all of them medial to the superior temporal lines and temporalis muscle attachments. The rostral two burr holes were placed just basal from the posterior interocular line, the basal two burr holes were placed just rostral to the lambdoid suture (and transverse sinuses) on both sides, respectively, and the middle two burr holes were placed in the line between the basal and rostral burr holes (Figure 1).

A laparotomy was made for the corresponding presentation of the peripheral veins and arteries (superior mesenteric vein, inferior caval vein, superior mesenteric artery, inferior mesenteric artery, inferior anterior pancreaticoduodenal artery, and abdominal aorta) and intestinal organs, and recording with a camera attached to a VMS-004 Discovery Deluxe USB microscope (Veho, Denver, CO, USA) was performed until the end of the experiment, and assessed after 5, 15, and 30 min of ligation.

To verify presentation of the activated collateral pathways in the rats that had an occluded superior mesenteric artery, the number of rats that exhibited a filled superior mesenteric artery distal from ligation and presented a superior and inferior anterior pancreaticoduodenal artery in mutual contact with the corresponding vein and that presented a filled inferior mesenteric artery and its ramification were recorded.

In general, the rat vessels were identified as described [49,50].

### 2.4. Angiography

Angiography was performed in rats with ligation of the superior mesenteric artery at 15 min post-ligation using a C-VISION PLUS fluoroscopy unit (Shimadzu, Kyoto, Japan) [19,24,25]. One milliliter, for 45 s, of warmed Omnipaque 350 (iohexol) non-ionic contrast medium (GE Healthcare, Chicago, IL, USA) was injected into the abdominal aorta at the level of the lumbar arteries. The contrast medium was visualized in real-time to ensure adequate filling. A subtraction mode was used to record the images at 14 frames per second. At 15 min post-ligation, angiograms were taken, captured, and digitized into files on a personal computer, and were analyzed using ISSA image software (Vamstec, Zagreb, Croatia). Angiography assessment included rats with full presentation of the superior mesenteric artery, distal from occlusion.

### 2.5. Superior Sagittal Sinus, Portal and Inferior Caval Vein, and Abdominal Aorta Pressure Recording

As described previously [19,24,25], recordings were made in a deeply anesthetized state after a cannula (BD Neoflon™ Cannula, Eysins, Switzerland) was connected to a pressure transducer (78534C MONITOR/TERMINAL; Hewlett Packard, Palo Alto, CA, USA) inserted into the superior sagittal sinus, portal vein, inferior vena cava, and abdominal aorta at the level of the bifurcation at 30 min post-ligation after 5 min of recording. For superior sagittal sinus pressure recording, we made a single burr hole in the rostral part of the sagittal suture, above the superior sagittal sinus (Figure 1), and cannulated the superior sagittal sinus anterior part by Braun intravenous cannulas. Then, we laparatomized rats for portal vein, inferior vena cava, and abdominal aorta pressure recording.

Notably, normal rats exhibited a superior sagittal sinus pressure of −24–27 mmHg and a superior mesenteric pressure and portal pressure of 3–5 mmHg, which is similar to that of the inferior vena cava, though with at least 1 mmHg higher values in the portal vein. By contrast, abdominal aorta blood pressure values were 100–120 mm Hg at the level of the bifurcation [19,24,25].

### 2.6. ECG Recording

ECGs were recorded continuously in deeply anesthetized rats for all three main leads by positioning stainless-steel electrodes on all four limbs using an ECG monitor with a 2090 programmer (Medtronic, Fridley, MI, USA) connected to a Waverunner LT342 digital oscilloscope (LeCroy, New York, NY, USA) after 30 min of ligation. This arrangement enabled precise recordings, measurements, and analysis of ECG parameters [19,24,25].

### 2.7. Thrombus Assessment

After being euthanized, the superior sagittal sinus, and, peripherally, the portal vein, inferior caval vein, superior mesenteric vein, lienal vein, and superior mesenteric artery were removed from the rats, and clots were weighed [19,24,25].

### 2.8. Brain Volume and Vessels Presentation Proportional with the Change of the Brain or Vessel Surface Area

The presentation of the brain, and peripheral veins (superior mesenteric and inferior caval) and arteries (abdominal aorta, inferior mesenteric artery, and superior mesenteric artery, part proximal to ligation and part distal from ligation) were recorded in deeply anaesthetized rats, with a camera attached to a VMS-004 Discovery Deluxe USB microscope (Veho, Denver, CO, USA), before the procedure in normal rats, and then, in rats with a ligated superior mesenteric vein after the procedure, before and after therapy, as well as after 5, 15, and 30 min of ligation before sacrifice. The borders of the brain or veins or arteries in the photograph were marked using ImageJ computer software and then, the surface area (in pixels) of the brain or veins was measured using a measuring function. This was done with brain photographs before the application and at intervals after the application for both control and treated animals. In the rats with an occluded superior mesenteric artery, the brain or vein or artery area before application was marked as 100% and the ratio of each subsequent brain area to the first area was calculated ($\frac{A_{2}}{A_{1}}$). Starting from square-cube law Equations (1,2), an equation for the change of the brain volume proportional to the change of the brain surface area (6) was derived. In Equations (1)–(5), *l* is defined as any arbitrary one-dimensional length of the brain (for example, the rostro-caudal length of the brain), used only for defining the one-dimensional proportion (*l*_2_/*l*_1_) between two observed brains and as an inter-factor (and because of this, it was not measured (6)) to derive the final expression (6). The procedure was as follows:

(1) $$A_{2}=A_{1}\times {\left(\frac{l_{2}}{l_{1}}\right)}^{2}$$

(square-cube law),

(2) $$V_{2}=V_{1}\times {\left(\frac{l_{2}}{l_{1}}\right)}^{3}$$

(square-cube law),

(3) $$\frac{A_{2}}{A_{1}}={\left(\frac{l_{2}}{l_{1}}\right)}^{2}$$

(from (1), after dividing both sides by *A*_1_),

(4) $$\frac{l_{2}}{l_{1}}=\sqrt{\frac{A_{2}}{A_{1}}}$$

(from (3), after taking the square root of both sides),

(5) $$\frac{V_{2}}{V_{1}}={\left(\frac{l_{2}}{l_{1}}\right)}^{3}$$

((from (2), after dividing both sides by *V*_1_),

(6) $$\frac{V_{2}}{V_{1}}={\left(\sqrt{\frac{A_{2}}{A_{1}}}\;\right)}^{3}$$

(after incorporating Equation (4) into Equation (5)).

### 2.9. Presentation of Stomach, Duodenum, and Gastrointestinal Serosal Disturbances

The presentation of gross lesions in the gastrointestinal tract and serosal disturbances were recorded in deeply anaesthetized rats with a camera attached to a VMS-004 Discovery Deluxe USB microscope (Veho, Denver, CO, USA). At 30 min post-ligation, we assessed hemorrhagic congestive areas in the stomach, duodenum, jejunum, cecum, and ascending colon (sum of the longest diameters, mm). Serosal disturbances (hemorrhage, vessels ramification, arterial filling, congestion) were assessed in the jejunum, cecum, and ascending colon and scored 0–4 as follows. We scored the bleeding on the intestinal surface as follows: 0 = no bleeding; 1 = barely indicated bleeding (diameter of the hematoma on the intestinal surface <1 mm); 2 = mild bleeding (diameter of the hematoma on the intestinal surface >1 mm–2 mm); 3 = moderate bleeding (diameter of the hematoma on the intestinal surface >2 mm–4 mm); 4 = intense bleeding (diameter of the hematoma on the intestinal surface >4 mm); 5 = very intense bleeding (flowing bleeding); venous congestion was scored as follows: 0 = venous congestion not present; 1 = barely indicated venous congestion (vein thickness up to 0.5 mm); 2 = mild venous congestion (vein thickness >0.5 mm–1 mm); 3 = moderate venous congestion (vein thickness >1 mm–2 mm); 4 = intense venous congestion (vein thickness >2 mm–2.5 mm); 5 = massive venous congestion (vein thickness >2.5 mm). Arterial filling was scored as follows: 0 = arterial filling not noticeable; 1 = barely indicated arterial filling (artery thickness up to 0.5 mm); 2 = mild arterial filling (artery thickness >0.5 mm–0.75 mm); 3 = moderate arterial filling (artery thickness >0.75 mm–2 mm); 4 = intensive arterial filling (artery thickness >2 mm–2.5 mm); 5 = massive arterial filling (artery thickness >2.5 mm). Arterial ramification was measured as follows: 0 = no noticeable ramification; 1 = barely indicated arterial ramification (2 visible branches); 2 = mild arterial ramification (3 visible branches); 3 = moderate arterial ramification (4 visible branches); 4 = intensive arterial ramification (5 visible branches); 5 = massive arterial ramification (6 or more visible branches).

### 2.10. Bilirubin and Enzyme Activity

To determine the serum levels of aspartate transaminase (AST), alanine transaminase (ALT, IU/L), and total bilirubin (μmol/L), blood samples were collected immediately after euthanasia and were centrifuged for 15 min at 3000 rpm. All tests were performed using an Olympus AU2700 analyzer with the original test reagents (Olympus Diagnostics, Lismeehan, Ireland) [24,25]. However, since there was no increase in bilirubin, the data are not shown [19,24,25].

### 2.11. Microscopy

Tissue specimens from the brain, liver, kidney, spleen, stomach, duodenum, jejunum, ascending colon, lungs, and heart were obtained from rats with superior mesenteric artery ligation after 30 min of ligation. These were fixed in buffered formalin (pH 7.4) for 24 h, dehydrated, and embedded in paraffin wax. The samples were stained with hematoxylin-eosin. Tissue injury was evaluated microscopically by a blinded examiner. Specifically, the brains were dissected using a coronal section with a mandatory 3 sections according to NTP-7, Level 3 and 6, due to neuroanatomic subsites being present in certain brain sections [51]. At NTP-7 Level 3, we observed an area of the fronto-parietal cortex, hippocampus, thalamus, and hypothalamus. At NTP-7 Level 6, we analyzed the cerebellar cortex morphology. Brain coronal blocks were embedded in paraffin, sectioned at 4 μm, stained with H&E, and evaluated by light microscopy using neuropathological scoring.

### 2.12. Brain Histology

Brain injury in different regions was evaluated using a semiquantitative neuropathological scoring system as described [52] (Table 1), providing a common score of 0–8, where grade 0 indicates no histopathologic damage.

### 2.13. Lung Histology

The following scoring system was used to grade the degree of lung injury in lung tissue analysis. Features were focal thickening of the alveolar membranes, congestion, pulmonary edema, intra-alveolar hemorrhage, interstitial neutrophil infiltration, and intra-alveolar neutrophil infiltration. Each feature was assigned a score from 0 to 3 based on its absence (0) or presence of a mild (1), moderate (2), or severe (3) degree, and a final histology score was determined [53].

### 2.14. Renal, Liver, and Heart Histology

Renal injury was based on degeneration of the Bowman space and glomeruli, degeneration of the proximal and distal tubule, vascular congestion, and interstitial edema. The criteria for liver injury were vacuolization of hepatocytes and pyknotic hepatocyte nuclei, activation of Kupffer cells, and enlargement of sinusoids. Each specimen was scored using a scale ranging from 0–3 (0 = none; 1 = mild; 2 = moderate; and 3 = severe), and for each criterion, a final histology score was determined [54,55]. Myocardium were graded according to the severity of congestion. Each specimen was scored using a scale ranging from 0–3 (0 = none; 1 = mild; 2 = moderate; and 3 = severe) to determine a final histology score.

### 2.15. Intestinal Histology

A histologic scoring scale adapted from Chui et al. [56] was used for tissue scoring on a scale of 0 to 5 (normal to severe) in three categories (mucosal injury, inflammation, hyperemia/hemorrhage) for a total score of 0 to 15 as described by Lane et al. [57].

Morphologic features of mucosal injury were based on different grades of epithelia lifting, villi denudation, and necrosis; grades of inflammation were graded from focal to diffuse according to lamina propria infiltration or subendothelial infiltration, and hyperemia/hemorrhage graded from focal to diffuse according to lamina propria or subendothelial localization.

### 2.16. Oxidative Stress

At the end of the experiment (at 30 min), oxidative stress in the collected tissue samples (plasma, ICV) was assessed by quantifying thiobarbituric acid-reactive species (TBARS) as malondialdehyde (MDA) (19, 22, 23, 26). The tissue samples were homogenized in PBS (pH 7.4) containing 0.1mM butylated hydroxytoluene (BHT) (TissueRuptor, Qiagen, Germantown, MD, USA) and sonicated for 30 s in an ice bath (Ultrasonic bath, Branson, MI, USA). Trichloroacetic acid (TCA, 10%) was added to the homogenate, the mixture was centrifuged at 3000 rpm for 5 min, and the supernatant was collected. Then, 1% TBA was added, and the samples were boiled (95 °C, 60 min). The tubes were then kept on ice for 10 min. Following centrifugation (14,000 rpm, 10 min), the absorbance of the mixture at a wavelength of 532 nm was determined.

The concentration of MDA was read from a standard calibration curve plotted using 1,1,3,3′-tetraethoxy propane (TEP). The extent of lipid peroxidation is expressed as MDA using a molar extinction coefficient for MDA of 1.56 × 105 mol/L/cm. The protein concentration was determined using a commercial kit. The results are expressed in nmol/g of protein.

### 2.17. Statistical Analysis

Statistical analysis was performed by parametric one-way analysis of variance (ANOVA), with a post-hoc Newman–Keuls test and non-parametric Kruskal–Wallis test and subsequently the Mann–Whitney U test to compare groups. Values are presented as the mean ± standard deviation (SD) and as the minimum/median/maximum. To compare the frequency difference between groups, the chi-square test or Fischer’s exact test was used. *p* < 0.05 was considered statistically significant.

## 3. Results

**Figure 2:**
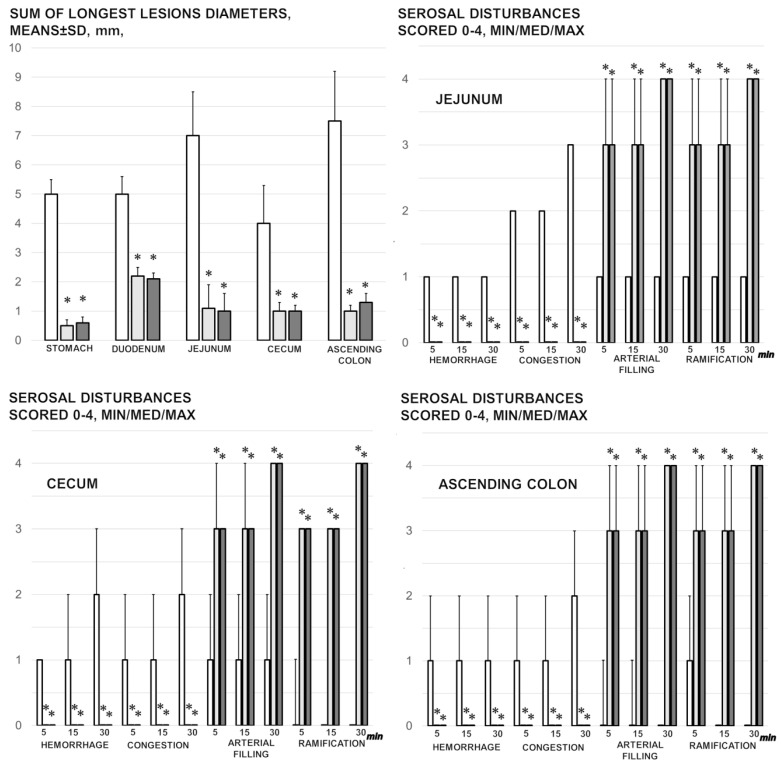
Mucosal lesions in the stomach, duodenum, jejunum, cecum, and ascending colon (sum of the longest lesions’ diameters, mm, means ± SD, mm, at 30 min of ligation), and the serosal disturbance in the stomach, duodenum, jejunum, cecum, and ascending colon, as hemorrhage, congestion, arterial filling, and ramification, scored 0–4, min/med/max) at 1, 5, 15, and 30 min after medication application (10 µg/kg BPC 157 (light gray bars), 10 ng/kg BPC 157 (dark gray bars), or 5 mL/kg saline (white bars). * *p* ˂ 0.05, at least, vs. control.

**Figure 3:**
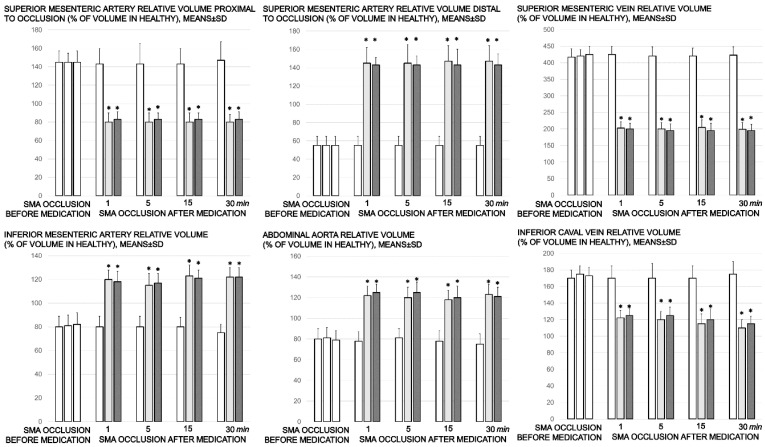
Proportional change of the vessel surface area assessed for failure of peripheral vessels’ development, as % of volume in healthy, means ± SD, mm), abdominal aorta, inferior mesenteric artery, inferior caval vein, and superior mesenteric artery relative volume at the time of the occlusion, proximal to ligation and distal to ligation, before medication application (SMA occlusion before medication) (white bars) and at 1, 5, 15, and 30 min after medication application (10 µg/kg BPC 157 (light gray bars), 10 ng/kg BPC 157 (dark gray bars), or 5mL/kg saline (white bars) (SMA occlusion after medication). All values significantly different (*p* ˂ 0.05, at least) vs. healthy values (100%); * *p* ˂ 0.05, at least, vs. corresponding control.

**Figure 4:**
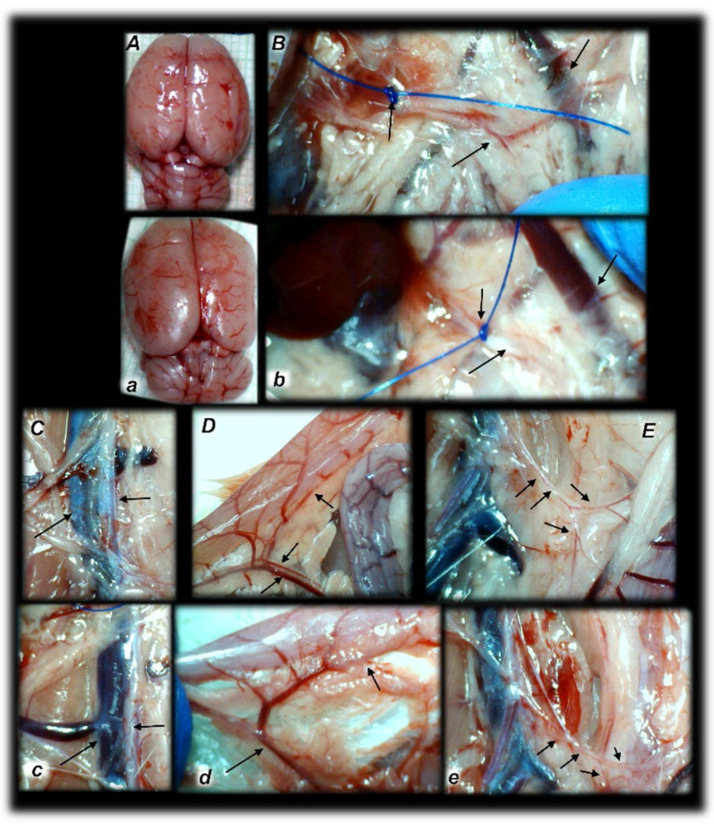
Rats with an occluded superior mesenteric artery and that received saline (small letters, **a**–**e**) or BPC 157 therapy (capitals, **A**–**E**) showed quite distinctive presentation of the brain at the end of the experiments (**a**,**A**), as well as the superior mesenteric artery (**b**,**B**), inferior caval vein and aorta (**c**,**C**), inferior anterior pancreaticoduodenal artery (**d**,**D**), and inferior mesenteric artery and its ramification (**e**,**E**) soon after mediation application. Arrows indicate important points. Arrows show ligation (**b**,**B**), and the distal part of the superior mesenteric artery, filled with blood (**B**) or thin and empty in controls (**b**), in addition to the superior mesenteric vein, congested (controls, **b**) and non-congested (BPC 157-rats, **B**). Arrows show the congested inferior caval vein and thin abdominal aorta (controls, **c**) and non-congested inferior caval vein and abdominal aorta with a maintained volume (BPC 157-rats, **C**). Arrows show the congested inferior anterior pancreaticoduodenal vein with no concurrent presentation of the inferior anterior pancreaticoduodenal artery along with the vein, and an apparent gape, and no communication with the superior anterior pancreaticoduodenal artery (controls, **d**), in contrast to the non-congested inferior anterior pancreaticoduodenal vein with clear presentation of the inferior anterior pancreaticoduodenal artery along with the vein, and clear communication with the superior anterior pancreaticoduodenal artery (BPC 157-treated rats, **D**). Arrows show the empty and thin inferior mesenteric artery (controls, **e**), and the inferior mesenteric artery filled with blood and its ramification (BPC 157-rats, **E**). The camera was attached to a VMS-004 Discovery Deluxe USB microscope (Veho, Denver, CO, USA).

**Figure 5:**
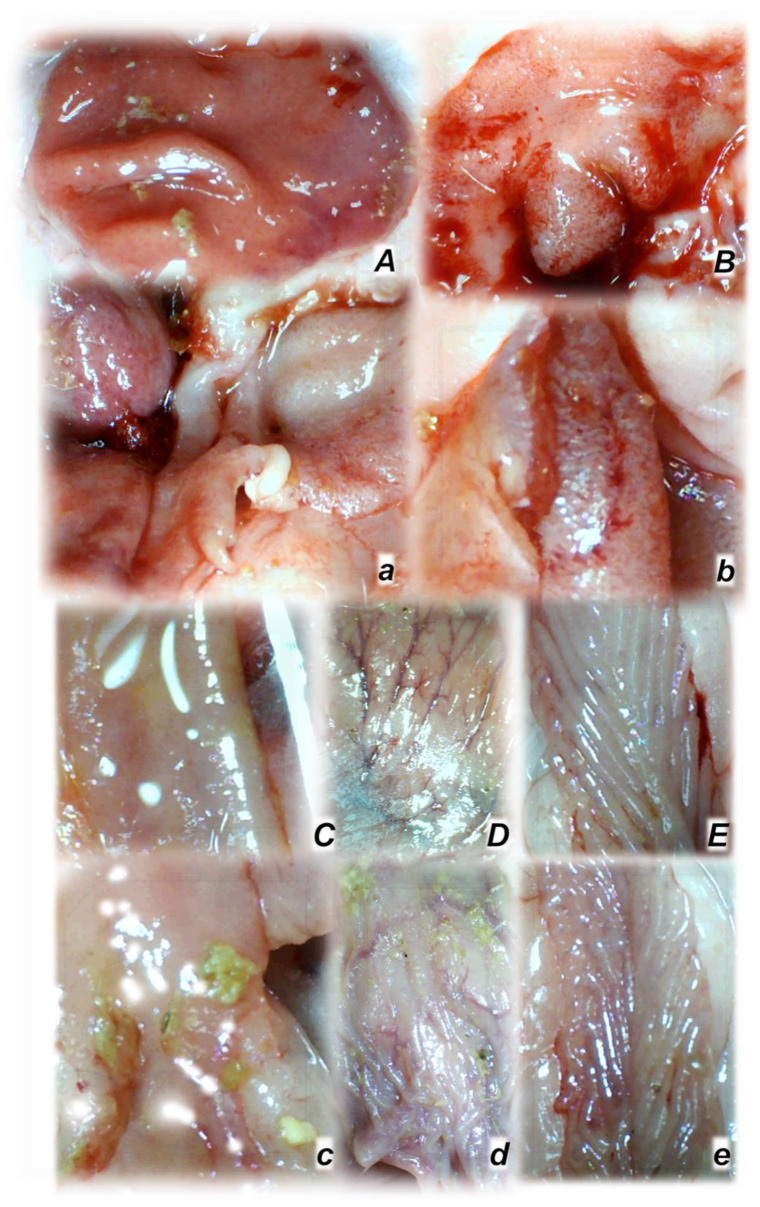
Rats with an occluded superior mesenteric artery and that received saline (small letters, **a**–**e**) or BPC 157 therapy (capitals, **A**–**E**) showed quite a distinctive mucosal presentation of the stomach (**a**,**A**), duodenum (**b**,**B**), jejunum (**c**,**C**), cecum (**d**,**D**), and ascending colon (**e**,**E**) at the end of the experiments, with evident lesions in controls (low) and preserved mucosa integrity in BPC 157-treated rats (upper). The camera was attached to a VMS-004 Discovery Deluxe USB microscope (Veho, Denver, CO, USA).

**Figure 6:**
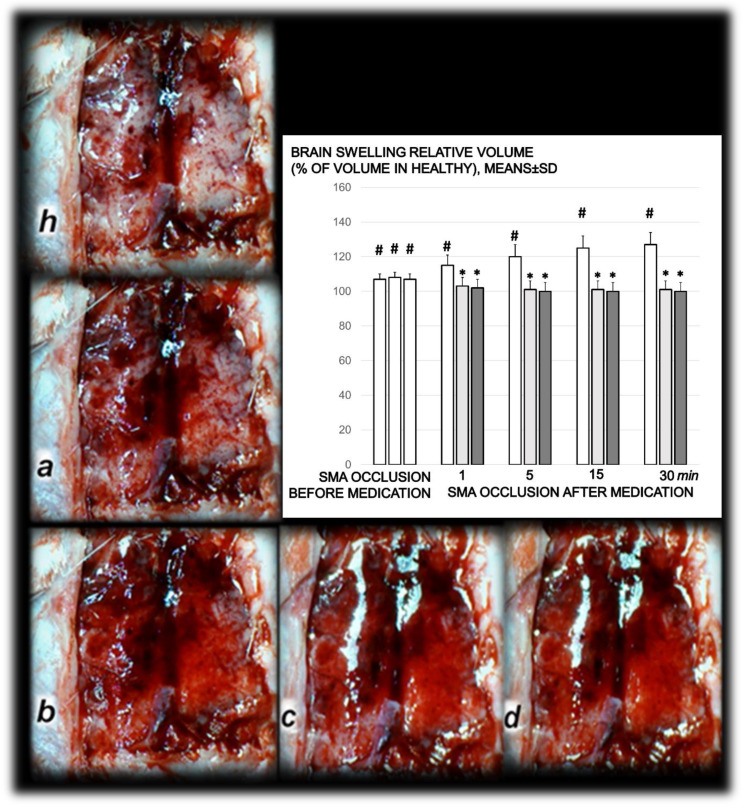
Timeline of the brain presentation in rats after complete calvariectomy: normal brain before superior mesenteric artery ligation (healthy, **h**), brain swelling immediately after occlusion of the superior mesenteric artery (**a**), and after BPC 157 therapy, a gradual decrease of the swelling, immediately after therapy (**b**), and at 5 min (**c**) and 15 min (**d**) thereafter. The camera was attached to a VMS-004 Discovery Deluxe USB microscope (Veho, Denver, CO, USA). Proportional change of the brain surface area assessed for the brain-swelling recording, as % of volume in healthy means ± SD, mm), brain, abdominal aorta, inferior mesenteric artery, inferior caval vein, and superior mesenteric artery relative to the volume at the time of the occlusion (SMA occlusion before medication) (white bars) and at 1, 5, 15, and 30 min after medication application (10 µg/kg BPC 157 (light gray bars), 10 ng/kg BPC 157 (dark gray bars), or 5 mL/kg saline (white bars) (SMA occlusion after medication). # *p* ˂ 0.05, at least, vs. healthy values (100%); * *p* ˂ 0.05, at least, vs. corresponding control.

**Figure 7:**
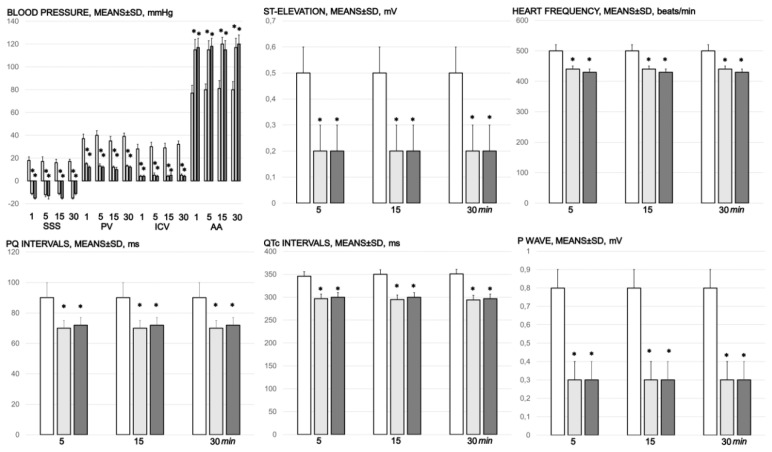
Blood pressure (mmHg) in the superior sagittal sinus (SSS), portal vein (PV), inferior caval vein (ICV), and abdominal aorta (AA), and ECG disturbance, ST elevation (mV), heart frequency (beats/min), PQ intervals (ms), QTc intervals (ms), and *p* wave amplitude (mV), mm, means ± SD) at 1, 5, 15, and 30 min after medication application (10 µg/kg BPC 157 (light gray bars), 10 ng/kg BPC 157 (dark gray bars), or 5 mL/kg saline (white bars). * *p* ˂ 0.05, at least, vs. control.

**Figure 8:**
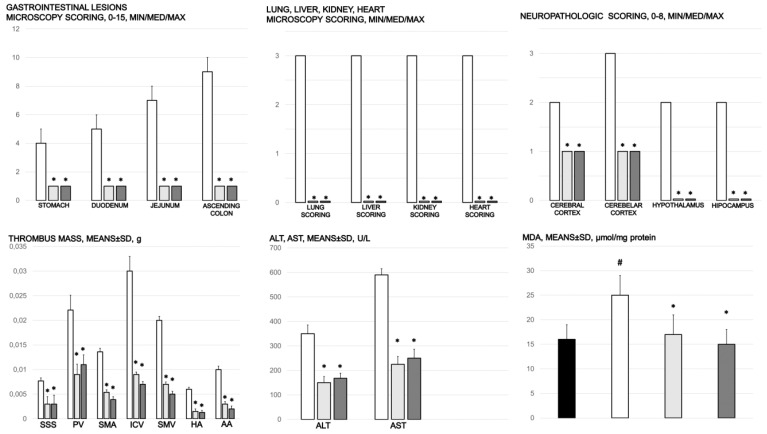
Gastrointestinal lesions (scored 0–15), scoring of the lesions in the lung (0–18), liver (0–9), kidney (0–12), heart (0–3), and brain (cerebral cortex, cerebellar cortex min/med/max thrombus mass (g)) in the superior sagittal sinus (SSS), portal vein (PV), superior mesenteric artery (SMA), inferior caval vein (ICV), superior mesenteric vein (SMV), hepatic artery (HA), and abdominal aorta (AA). Blood pressure (mmHg) in the superior sagittal sinus (SSS), portal vein (PV), inferior caval vein (ICV), and abdominal aorta (AA), means ± SD, serum enzymes (ALT, AST) values (U/L), means ± SD, and MDA values (µmol/mg protein) in cecum, means ± SD, at the end of the experiment in the rats with occlusion of the superior mesenteric artery and that received medication application (10 µg/kg BPC 157 (light gray bars), 10 ng/kg BPC 157 (dark gray bars), or 5mL/kg saline (white bars). * *p* ˂ 0.05, at least, vs. control.

**Figure 9:**
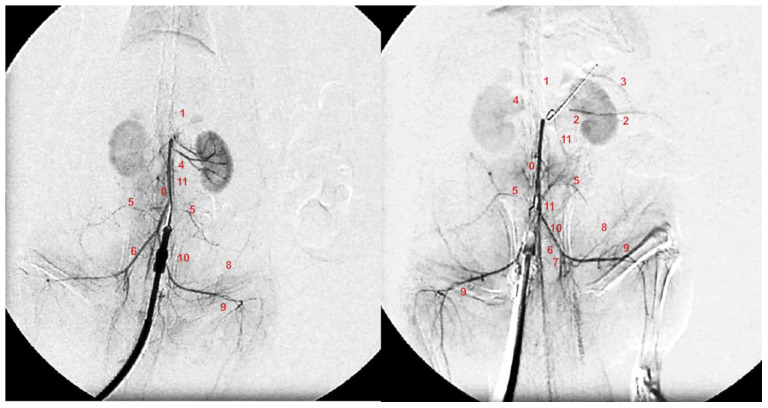
Angiography was performed in rats with ligation of the superior mesenteric artery at 15 min post-ligation, using a C-VISION PLUS fluoroscopy unit (Shimadzu, Japan). One milliliter, for 45 s, of warmed Omnipaque 350 (iohexol) non-ionic contrast medium (GE Healthcare, USA) was injected into the abdominal aorta at the level of the lumbar arteries. Presentation of the abdominal aorta (0), coeliac trunk (1), superior mesenteric artery (2), inferior anterior pancreaticoduodenal artery (3), renal artery (4), iliolumbar artery (5), common iliac artery (6), internal iliac artery (7), caudal epigastric artery (8), iliacofemoral artery (9), testicular artery (10), and inferior mesenteric artery (11) in rats (Low et al., 2016) with occlusion of the superior mesenteric artery, immediately after they received medication application (10 µg/kg BPC 157 (**right**) or 5 mL/kg saline (**left**) at 15 min of ligation.

**Figure 10:**
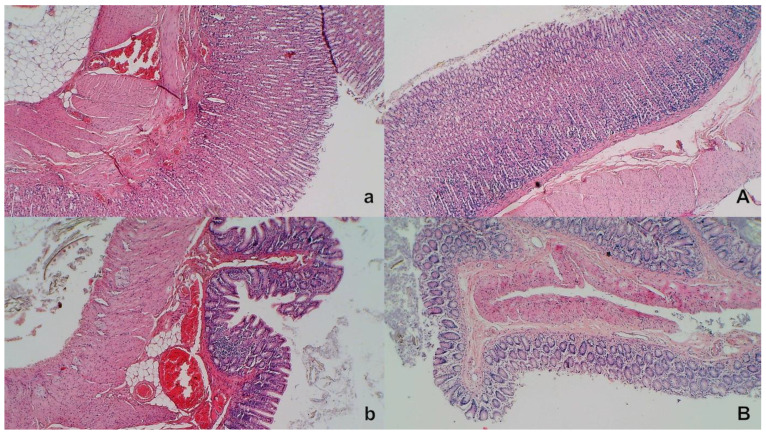
Stomach and duodenal injury (HE x100). Control rats show marked transmural congestion within the lamina propria of the stomach (**a**, left, upper) and duodenum (**b**, left, low). Blunt duodenal villi and mild hyperplasia of the crypts. Contrarily, BPC 157 rats exhibit only mild congestion, with preserved stomach (**A**, right, upper) and duodenal (**B**, right, low) architecture of the mucosa.

**Figure 11:**
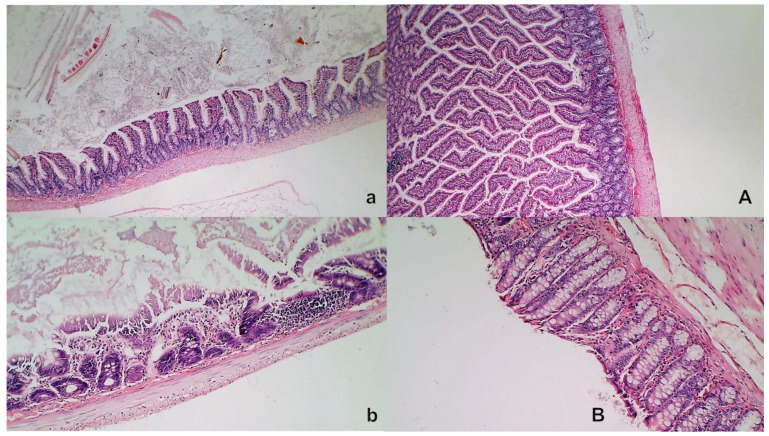
Intestinal injury (HE, ×100 (upper), ×200 (low)). Controls presented with moderately severe mucosal injury with a reduction of intestinal villi; more severe mucosal injury with lumen dilatation of the colon and a reduction of crypts (**a**,**b**, left). Contrarily, BPC 157-treated rats exhibited only mild congestion, with preserved mucosal intestinal architecture (**A**,**B**, right).

**Figure 12:**
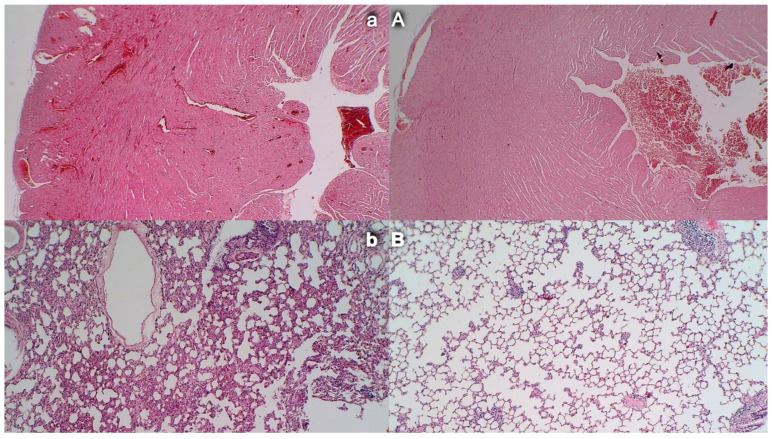
Congestion and hemorrhage of the heart and lung. (HE, ×100). Control rats exhibit congestion within the myocardium (**a**, left, upper) and lung septa along with intralveolar hemorrhage; lung edema and focal accumulation of neutrophils within septa (**b**, left, low). Contrarily, no heart congestion (**A**, right, upper) and no lung congestion and no intralveolar hemorrhage (**B**, right, low) were observed in BPC 157-treated rats.

**Figure 13:**
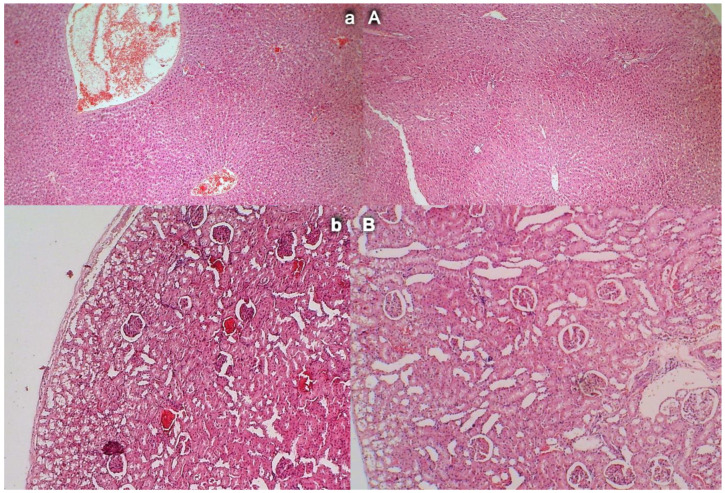
Liver and kidney injury (HE, ×100). In liver parenchyma, control rats showed congestion and dilatation of the sinusoids and central veins (**a**, left, upper) while BPC 157 rats showed no changes in liver parenchyma (**A**, right, upper). Controls showed congestion of the renal parenchyma along with congestion of the glomeruli (**b**, left, low); BPC 157 rats showed no changes in renal parenchyma (**B**, right, low).

**Figure 14:**
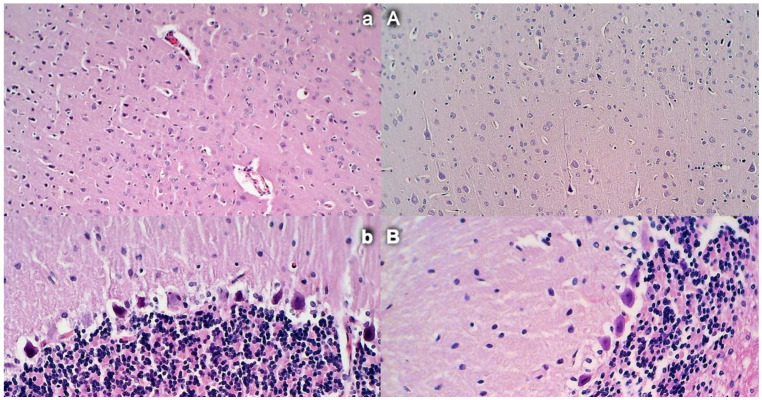
Brain histology. Control rats presented increased edema and congestion and an increased number of karyopyknotic cells in the cerebral (**a**) (HE, ×200) and cerebellar cortex (**b**) (HE, ×400). Contrarily, BPC 157-treated rats exhibited a few karyopyknotic neuronal cells in the cerebral (**A**) (HE, ×200) and cerebellar cortex (**B**) (HE, ×400).

**Figure 15:**
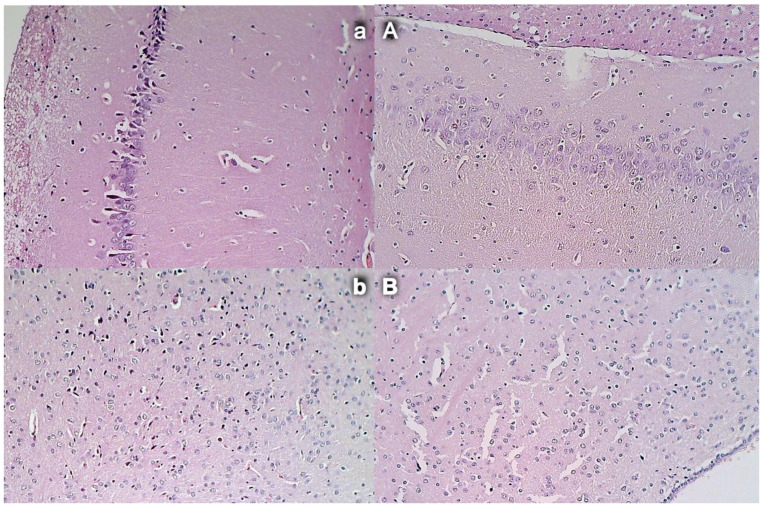
Brain histology: Control rats presented increased edema and congestion and an increased number of karyopyknotic cells in the hippocampus cortex (**a**) and hypothalamus (**b**), (HE, ×200). Contrarily, BPC 157-treated rats exhibited a few karyopyknotic neuronal cells in the hippocampus cortex (**A**) and hypothalamus (**B**) (HE, ×200).

BPC 157 therapy (both µg and ng regimens) counteracted multiple organ dysfunction syndrome in rats that have an occluded superior mesenteric artery (Figure 2, Figure 3, Figure 4, Figure 5, Figure 6, Figure 7, Figure 8, Figure 9, Figure 10, Figure 11, Figure 12, Figure 13, Figure 14 and Figure 15), providing beneficial effects (i.e., eliminated oxidative stress). Once occlusion of the superior mesenteric artery occurred, the full perilous syndrome instantly appeared. The therapy effect manifestations simultaneously occurred. Peripherally, these include activation of the collateral pathways, maintenance of heart function, decrease of organ lesions both grossly and microscopically, and attenuation/elimination of portal and caval hypertension and aortal hypotension. Centrally, an immediate decrease of brain swelling, attenuation of brain lesions, and decrease of intracranial hypertension (i.e., superior sagittal sinus hypertension) occurred. Peripherally and centrally, thrombosis formation was attenuated (Figure 2, Figure 3, Figure 4, Figure 5, Figure 6, Figure 7, Figure 8, Figure 9, Figure 10, Figure 11, Figure 12, Figure 13, Figure 14 and Figure 15).

This was shown by careful assessments. Consistent gross recordings were observed, including swollen brain and peripheral vessels from the relative volume recording (Figure 2, Figure 3, Figure 4, Figure 5 and Figure 6); blood pressure assessment in the superior sagittal sinus, portal and caval vein, and abdominal aorta (Figure 7); ECG recording (Figure 7), thrombosis assessment in the arteries (hepatic and superior mesenteric artery, abdominal aorta) and veins (superior sagittal sinus, portal, inferior caval and superior mesenteric vein) (Figure 8), angiography (Figure 9), microscopy assessment (Figure 8, Figure 10, Figure 11, Figure 12, Figure 13, Figure 14 and Figure 15), serum enzymes (Figure 8), and oxidative stress recording (Figure 8). Therapy implies the reversal of the full injury course, including µg and ng regimens. Likely, this may be the effect of rapid activation of alternative bypassing pathways, identified by direct recording and confirmed by angiography (i.e., Figure 4 and Figure 9).

### 3.1. Gross Gastrointestinal Lesions

Without therapy, rats with a ligated superior mesenteric artery regularly exhibited severe lesions in the whole gastrointestinal tract, with ascending severity from the stomach toward the ascending colon (Figure 2 and Figure 5). Regularly, throughout the gastrointestinal tract, bleeding mucosal lesions were observed, as well as at the serosa, and the rats with a ligated superior mesenteric artery presented considerable hemorrhage and congestion, failed arterial filling, and lack of ramification (thereby lacking interconnection between the arcade vessels). Contrarily, BPC 157 rats presented markedly less mucosal lesions, and considerably less hemorrhage and congestion at the serosa, preserved arterial filling, and advanced ramification seen as small collaterals rapidly presented and fully perfused interconnections between the neighboring vessels.

### 3.2. Brain Swelling and Peripheral Vessel Failure Presentation and Counteraction

Proportional change of the vessel or brain surface area was used for the assessment of the failure of peripheral vessels’ development as well as brain swelling recording (Figure 3 and Figure 6).

Without therapy, ligation of the superior mesenteric artery rapidly induces peripheral vessels’ failure (proportional with the change of the healthy surface area), and the superior mesenteric vein and inferior caval vein volume immediately revealed a considerable increase, and congestion. Contrarily, the abdominal aorta and inferior mesenteric artery appeared thin. The superior mesenteric artery part proximal to the ligation appeared congested while the part distal to the ligation was very thin (Figure 3 and Figure 4).

Administration of BPC 157 therapy completely reversed the vessel presentation (Figure 3 and Figure 4), as part of the organized bypassing pathways compensating the occlusion of the superior mesenteric artery. The superior mesenteric artery part proximal to the ligation appeared thin while the part distal to the ligation was filled with blood and showed an increased relative volume (Figure 3 and Figure 4). Likewise, the abdominal aorta and inferior mesenteric artery appeared with an increased relative volume. The considerable increase of the relative volume of the superior mesenteric vein and inferior caval was markedly decreased and the volume approached the values observed for the healthy rats.

In addition to the recovered presentation of the superior mesenteric artery, direct recording revealed that only rats that received BPC 157 therapy presented the inferior and superior pancreaticoduodenal arteries along with the vein. Close contact between these arteries may bridge occlusion at the superior mesenteric artery. Similarly, in BPC 157 rats, an inferior mesenteric artery filled with blood with evident ramification (i.e., left colic artery toward middle colic artery) was observed to bridge occlusion at the superior mesenteric artery from the other side (Fisher exact test *p* ˂ 0.05, at least vs. control).

Additionally, at the same time, without therapy, increased swelling of the brains of rats with an occluded superior mesenteric artery was observed (Figure 6). As a consistent and prominent effect, BPC 157 therapy rapidly attenuated brain swelling close to the normal pre-procedure values with the µg and ng regimens. This effect is in parallel with the effect on the peripheral blood vessels’ presentation.

### 3.3. Portal and Caval Vein and Abdominal Aorta and Superior Sagittal Sinus Pressure Recording

Without therapy, rats with an occluded superior mesenteric artery exhibited high portal hypertension, caval hypertension, and aortal hypotension. In addition, they exhibited intracranial hypertension, assessed as hypertension in the superior sagittal sinus (note, negative pressure in the superior sagittal sinus is immediately changed with the increased (positive) pressure) (Figure 7). Vice versa, with BPC 157 therapy of µg and ng regimens, the blood pressure disturbances were markedly mitigated. The severe portal and caval hypertension and aortal hypotension were rapidly attenuated or resolved, as well as intracranial (superior sagittal sinus) hypertension (i.e., the increased (positive) pressure in the superior sagittal sinus was immediately substituted with the negative pressure).

### 3.4. Thrombosis

Without therapy, thrombosis rapidly appeared in all vessels tested (i.e., superior sagittal sinus, portal, superior mesenteric, and inferior caval vein as well hepatic and superior mesenteric artery and abdominal aorta) in rats with an occluded superior mesenteric artery. BPC 157, given at 1 min of ligation, markedly counteracted and reversed thrombosis presentation in both arteries and veins (Figure 8).

### 3.5. Angiography

Without medication, abdominal aorta angiography showed poor presentation in the rats with a ligated superior mesenteric artery (Figure 9). Commonly, they showed a lack of activated collaterals, and no presentation of the occluded superior mesenteric artery was observed.

BPC 157 medication fully counteracted these disturbances. Angiography revealed in all BPC 157 rats, after occlusion, a filled distal part of the superior mesenteric artery (*p* ˂ 0.05, at least, vs. control), and activated collaterals, as may be seen with the presentation of the inferior mesenteric artery and inferior anterior pancreaticoduodenal artery.

### 3.6. ECG Recording

Tachycardia, increased *p* wave amplitude, prolonged QT intervals, and significant ST elevation appeared rapidly in rats with occluded superior mesenteric arteries, which were markedly counteracted by the BPC 157 regimens (Figure 2).

### 3.7. Enzymes

Serum ALT and AST values increased in controls; they were lower in rats treated with BPC 157 (Figure 8).

### 3.8. Microscopy

Microscopy presentation of BPC 157 therapy in rats with an occluded superior mesenteric artery confirmed the reversal of the noxious parameters. These were shown to be counteracted, peripherally and centrally (i.e., the brain swelling and failed vessels presentation, superior sagittal sinus, portal, and caval vein hypertension and abdominal aorta hypotension, thrombosis, arterial and venous, gastrointestinal tract lesions, ECG disturbances, increased serum enzymes and oxidative stress presentation).

Together, this may be a particular severe syndrome, such as ongoing Virchow’s triad peripherally and centrally, thus counteracting the potential of the given BPC 157 therapy.

The lesions may be superior mesenteric artery ligation specific, in terms of the cause–consequence relations, complete occlusion of the superior mesenteric artery by ligation, and no activation of the collateral pathways versus activation of the collateral pathways to bypass occlusion and reestablished blood flow.

### 3.9. Stomach, Duodenum, and Intestinal Injury

Without therapy, all of the rats with an occluded superior mesenteric artery exhibited marked transmural congestion within the stomach, duodenum, and small and large bowel wall, with an ascending sequence from the stomach to the large bowel (Figure 10 and Figure 11). Illustratively, the stomach dilated capillaries in the lamina propria. Within the small and large bowel mucosa, focal hemorrhage appeared in the lamina propria. Mild mucosal injury appeared with blunt duodenal villi and mild hyperplasia of the crypts while more severe mucosal injury with a reduction of intestinal villi and even more severe mucosal injury with lumen dilatation of the colon and reduction of crypts were observed. Contrarily, in rats with an occluded superior mesenteric artery that received BPC 157 therapy, most of these changes were not found. Only mild congestion was observed.

### 3.10. Heart Damage

Severe congestion within the myocardium was observed in controls with an occluded superior mesenteric artery while no changes within the myocardium were found in BPC 157-treated rats (Figure 12).

### 3.11. Lung Damage

Occlusion of the superior mesenteric artery commonly produced focal thickening of the alveolar membranes, lung congestion, pulmonary edema, intra-alveolar hemorrhage, and focal interstitial neutrophil infiltration in controls. Contrarily, BPC 157 rats presented only discrete lung congestion, with no other changes in lung parenchyma (Figure 12).

### 3.12. Liver Damage

Mild activation of Kupffer cells and severe enlargement of sinusoids with liver congestion appeared in controls with occlusion of the superior mesenteric artery (Figure 13). Contrarily, no changes appeared in liver parenchyma in BPC 157-treated rats.

### 3.13. Renal Damage

Occlusion of the superior mesenteric artery produced a mild degeneration of proximal tubules, severe vascular congestion, and mild interstitial edema (Figure 13). Contrarily, no changes were found in renal parenchyma in BPC 157-treated rats.

### 3.14. Brain Damage

Commonly, unless therapy was given, rats with an occluded superior mesenteric artery exhibited an increased number of karyopyknotic cells in all four region: cerebral and cerebellar cortex, hypothalamus, thalamus, hippocampus cortex, and hypothalamus/thalamus (Figure 14 and Figure 15). Neuropathologic changes in cerebral cortex areas revealed increased edema and congestion. Especially, karyopyknosis and degeneration of Purkinje cells of the cerebellar cortex and marked karyopyknosis of pyramidal cells of the hippocampus were observed. Contrarily, BPC 157 rats showed a few karyopyknotic neuronal cells in the analyzed neuroanatomic structures.

### 3.15. Oxidative Stress

Without medication, rats with a ligated superior mesenteric artery regularly showed increased MDA values (Figure 8). This was completely counteracted in the rats that received BPC 157 medication.

## 4. Discussion

BPC 157 therapy counteracts, acting both peripherally and centrally, multiple organ dysfunction syndrome in rats with occlusion of the superior mesenteric artery. Illustrating the rapid onset of the beneficial effect, grossly, considerable brain swelling is rapidly reversed upon BPC 157 application, and brain lesions markedly attenuated.

Of note, the observed therapy effect in the severely affected rats with an occluded superior mesenteric artery was a strong one as noted before in the Pringle maneuver, ischemia, reperfusion, and Budd–Chiari-syndrome [24,25] and other vessel occlusion studies [18,19,21,22,23]. To support this common conclusion, it should be noted that whatever may be the particular difference between the venous occlusion (Pringle maneuver, ischemia, reperfusion, and Budd–Chiari-syndrome) [24,25] and artery occlusion (superior mesenteric artery), multiple organ dysfunction syndrome shares a similar rapid onset and widespread presentation, and corresponds to each other. In addition, BPC 157 counteracted the intracranial (superior sagittal sinus) hypertension and brain lesions in the rats with an occluded superior mesenteric artery. Additionally, BPC 157 therapy counteracted oxidative stress; markedly mitigated hemorrhagic gastrointestinal tract lesions (in ascending severity from the stomach to the colon, lung, and liver), kidney congestion and hemorrhage, ECG disturbances and heart congestion, and arterial and venous thrombosis; and attenuated or eliminated portal and caval hypertension and aortal hypotension. Likewise, similar rapid activation of the vessels corresponds to the collateral pathways to bridge the occlusion at the superior mesenteric artery. In BPC 157 rats only, communication between the superior and inferior anterior pancreaticoduodenal artery appeared from one side, and artery presentation along with the corresponding veins were observed (vessels emptied/disappeared (i.e., in controls); refilled/reappeared (i.e., in BPC 157-treated rats)). In BPC 157 rats only, a filled inferior mesenteric artery and its further ramification appeared from the other side. In BPC 157 rats only, the recovery result appeared in the superior mesenteric artery filled with blood distal to the occlusion, and the findings were confirmed by angiography as well. The evidenced activated collateral pathways, obtained from the direct recording and angiography, may serve well to compensate, at least partly, the occlusion of the superior mesenteric artery.

Furthermore, we can suggest that this innate recovery requires activation through the specific essential collateral pathways depending on the occlusion injury [18,19,21,22,23,24,25]. Exemplified are the rats with the excluded part of the left colic artery and vein by two ligations, ischemic/reperfusion colitis [18] or the inferior caval vein syndrome (i.e., infrarenal ligation of the inferior vena cava) [19]. Receiving BPC 157 therapy induces rapid activation of a bypassing loop from the existing vessels (i.e., intestine arcade vessels network, or left ovarian vein) [18,19]. In the Pringle maneuver, the resolving bypassing porto-caval loop is through the inferior mesenteric vein [25]. In Budd–Chiari syndrome, the resolving bypassing inferior caval vein-left superior caval vein loop is through the azygos vein [25]. Additionally, counteracted duodenal venous congestion and lesions [21], ameliorated perforated cecum [22], bile duct ligation-induced liver cirrhosis [23], and reversed portal hypertension [23,24,25] have been observed. Recently, BPC 157’s beneficial effect, given in reperfusion, on brain lesions of stroke rats shows that BPC 157 therapy counteracts both early and delayed neural hippocampal damage, achieving full functional recovery [20]. Thus, these findings verify a common principle of Virchow’s triad situation that is commonly effectively relieved, centrally and peripherally, with the particular rapid activation of a specific bypassing loop [24,25]. There is always recovery through the essential collateral pathways, which are therefore specifically activated [18,19,21,22,23,24,25].

Consequently, as the additional resolving final point, the eliminated/attenuated thrombosis and stasis appeared in all vessels tested (i.e., superior sagittal sinus, portal, superior mesenteric, and inferior caval vein as well as hepatic and superior mesenteric artery and abdominal aorta). As also seen in the rat Pringle maneuver ischemia, reperfusion, and Budd–Chiari syndrome [19,24,25], these may appear as the positive initial point (quick endothelium rescue, operating collaterals, as common rapid cytoprotective response to injury). In support, BPC 157 also maintains pivotal platelet function [58]. Of note, BPC 157 decreases the formation of cloth after aortic termino-terminal anastomosis; in rats with a formed cloth obstructing more than one-third of the aortic lumen, no cloth could be seen at the anastomosis site after BPC 157 administration [30].

Furthermore, heart dysfunction, as seen in therapy of rat Pringle maneuver ischemia, reperfusion, and Budd–Chiari syndrome [24,25], may be important in the multiple mutual cause–consequence relationships in vascular occlusion-induced syndrome presentation in rats with occluded superior mesenteric arteries. Occlusion of the superior mesenteric artery is instant, and would immediately contribute to heart dysfunction and vice versa. Tachycardia, increased *p* wave amplitude, prolonged QT intervals, significant ST elevation, and severe myocardial congestion appeared rapidly in rats with occluded superior mesenteric arteries. In particular, right ventricular function [59,60] is known to be inversely correlated to a prolonged QT interval [60]. If not corrected, lung congestion appears as a common outcome (i.e., time-dependent and time-independent features that can be acute respiratory distress syndrome exudative-phase features), and acute lung injury is a primary component of multiple organ dysfunction syndromes triggered by intestinal ischemia-reperfusion, which results in high mortality and acute lung injury [61,62]. Then, an additional common outcome is liver failure (substantial congestion of the central vein as well as branches of the portal veins in portal triads), prominent portal and caval hypertension, and consequential gastrointestinal hemorrhagic lesions [24,25]. Vascular collapse can suddenly appear as seen with the congested inferior caval vein and superior mesenteric vein, and thin presentation of the abdominal aorta and inferior mesenteric artery, and finally the empty superior mesenteric artery. Likewise, it was pointed out that cardiac arrhythmias occur following a rapid increase in intracranial pressure, and vice versa [63].

Thereby, the additional important point may be mandatory, in that BPC 157 has strong cardioprotective and antiarrhythmic effects as demonstrated in the present and recent vascular occlusion studies [19,24,25]. Strong cardioprotective and antiarrhythmic effects were also evidenced in other studies [64,65,66,67,68]. Of note, BPC 157 prevented as well as reversed doxorubicine chronic heart failure [64] and various arrhythmias induced by different agents and noxious procedures [65,66,67,68], in particular prolonged QT intervals [68]. Likewise, as in the vascular occlusion studies [24,25], BPC 157 counteracted severe lesions that may be regularly induced by the different agents and noxious procedures in the lung [69,70,71], liver [1,72,73,74,75,76,77,78], and gastrointestinal tract [72,73,74,75,76,77]. Finally, BPC 157 attenuated brain lesions, induced by trauma, stroke, or various encephalopathies [20,72,73,74,75,76,77,79]. In addition, there is reduced malondialdehyde (MDA), even to the normal levels, as a confirmative result of both preserved and rescued intestinal mucosal integrity and artery integrity, and a free radical scavenger effect. This occurs as before in both ischemic and reperfusion conditions in the various tissues (i.e., brain, colon, duodenum, cecum, liver, and veins) and plasma [18,19,21,22,25,39,40,41].

Finally, from the central viewpoint, counteraction of the harmful effects of an occluded superior mesenteric artery in rats should consider that increased pressure in the superior sagittal sinus reflects an inability to drain venous blood adequately for a given cerebral blood inflow without raising the venous pressure, which suddenly becomes venous and intracranial hypertension. Additionally, it may be that occlusion of the superior mesenteric artery initiates increased abdominal pressure, and thereby increased intracranial pressure, which is mainly attributed to the transmission of pressure from the abdominal cavity to the venous system above the diaphragm, leading to a reduction of venous return from the brain [80,81]. Additionally, in response to increased abdominal pressure, there may be a vicious circle, as intracranial pressure is significantly increased, superior mesenteric artery flow is significantly decreased, perpetuating the injurious course [80,81]. Elevated intra-abdominal pressure produces cranial displacement of the diaphragm and increases intrathoracic pressure. This leads to high filling pressure of the right atrium, increased jugular vein pressure, and subsequent reduction of the venous return from the brain [80,81].

In conclusion, the therapy effect of BPC 157 administration in the occluded superior mesenteric artery rats and their recovery would summarize the initial background. We observed Robert’s stomach cytoprotection [26], endothelial lesions, abundant thrombi in superficial capillaries, and diffuse vascular stasis [82]; thereby, the Virchow triad, which should be rapidly counteracted, is used to achieve stomach cytoprotection [12]. From the cytoprotective theoretical viewpoint, this response that should be generalized [26], as it is the ultimate rapid beneficial effects of the stable gastric pentadecapeptide BPC 157 application and rapid activation of the collateral pathways, consistently obtained in the occluded superior mesenteric artery rats, as well as in other vascular occlusion-induced syndromes. Consequently, BPC 157 interacts with the NO system in various models and species [1,2,3,4,5,6,7,8,9,10,11,12,13,14,15,16,17]. As a specific result, this activates collateral vessel pathways [18,19,20,21,22,23,24,25]. It should be noted that BPC 157 may induce NO release of its own [83], even in the condition where L-arginine is not working [48]. Additionally, BPC 157’s curative effect is associated with several molecular pathways [15,16,19,20,31,42,43,44,45,46,47,84]. Illustratively, BPC 157 accelerates blood flow recovery and the vessel number in rats with hind limb ischemia by increased expression and internalization of vascular endothelial growth factor receptor 2 (VEGFR2) and activation of the VEGFR2-Akt-endothelial nitric oxide synthase (eNOS) signaling pathway [31,46], executed without the need for other ligands or shear stress [46]. The beneficial effects in rats with an occluded inferior caval vein are in conjunction with distinctive presentation in the vessels of the *Egr*, *Nos*, *Srf*, *Vegfr*, *Akt1*, *Plcɣ*, and *Kras* pathways [19]. It seems that this depends on whether the assessed vessels were blinded, obstructed (right ovarian vein and inferior caval vein), or present an alternative collateral pathway (i.e., left ovarian vein), which bypasses occlusion of the main vessel [19]. Finally, as an additional clue, BPC 157 may be a stabilizer of cellular junctions [16]. Via increased tight junction protein ZO-1 expression and transepithelial resistance [16], it significantly mitigated indomethacin-induced leaky gut syndrome [16]. Likewise, inhibition of the mRNA of inflammatory mediators (iNOS, IL-6, IFNγ, and TNF-α), increased expression of HSP 70 and 90, and antioxidant proteins, such as HO-1, NQO-1, glutathione reductase, glutathione peroxidase 2, and GST-pi, were also observed [16]. Additionally, to support BPC 157’s beneficial effect on brain lesions, a very recent demonstration in stroke rats showed that BPC 157 can restore recognition memory deficits along with the therapy effect [20]. At 24 and 72 h of reperfusion, the therapy counteracted both early and delayed neural hippocampal damage, achieving full functional recovery (Morris water maze test, inclined beam-walking test, lateral push test). mRNA expression studies at 1 and 24 h, provided strongly elevated (Egr1, Akt1, Kras, Src, Foxo, Srf, Vegfr2, Nos3, Nos1) and decreased (Nos2, Nfkb) gene expression (Mapk1 not activated), as a mechanism of how BPC 157 may act [20].

Thus, BPC 157 therapy rapidly recovered the superior mesenteric artery ligated rat, and raised the bypassing principle (it should be noted that it was impossible to adequately keep the control animals alive for a longer period, and thereby, the focus was on the period investigated). On the other hand, for BPC 157 application and occlusion of the superior mesenteric artery in rats, we should emphasize the general point that animal studies per se may be cautious regarding their results, although the role of the animal model is indispensable. Additionally, we should emphasize the relative paucity of BPC 157 clinical data [1,2,3,4,5,6,7,8,9,10,11,12,13,14,15,16,17]. However, it should be noted that BPC 157 was proved to be efficacious in the ulcerative colitis, both in clinical settings [85,86] as well as in experimental rat ischemic/reperfusion vascular ulcerative colitis studies [18], and it has a very safe profile (LD1 could be not achieved) [1,2,3,4,5,6,7,8,9,10,11,12,13,14,15,16,17], a point recently confirmed in a large study by Xu and collaborators [87]. Thus, while additional studies should be done, we can claim that rapidly activating alternative bypassing pathways overrides permanent ligation of the superior mesenteric artery, and provides therapeutic strategies that should be further evaluated.

## Figures and Tables

**Table 1:** The neuropathologic scores.

Brain Area	Grading	Percent Area Affected	Morphological Changes
Cerebral and cerebellar cortex, hypothalamus, thalamus, hippocampus	1	≤10	Small, patchy, complete or incomplete infarcts
	2	20–30	Partly confluent complete or incomplete infarcts
	3	40–60	Large confluent compete infarcts
	4	>75	In cortex; total disintegration of the tissue, in hypothalamus, thalamus, hippocampus; large complete infarcts
Cerebral and cerebellar cortex, hypothalamus, thalamus, hippocampus	1	≤20	A few karyopyknotic of neuronal cells
	2	50	Patchy areas of karyopyknotic areas
	3	75	More extensive of karyopyknotic areas
	4	100	Complete infarction

## Data Availability

The data presented in this study are available on request from the corresponding author.
